# SlimPort: Port-Driven High-Level Synthesis for Continuous-Flow Microfluidic Biochips

**DOI:** 10.3390/mi16050577

**Published:** 2025-05-14

**Authors:** Youlin Pan, Yanbo Xu, Ziyang Chen, Xing Huang, Genggeng Liu

**Affiliations:** 1 College of Computer and Data Science, Fuzhou University, Fuzhou 350116, China; panyoulin@163.com (Y.P.); pollystevens@163.com (Y.X.); czyeeric@126.com (Z.C.); 2 Engineering Research Center of Big Data Intelligence, Ministry of Education, Fuzhou 350116, China; 3 Fujian Provincial Key Laboratory of Network Computing and Intelligent Information Processing, Fuzhou 350116, China; 4 School of Computer Science, Northwestern Polytechnical University, Xi’an 710072, China; xing.huang@nwpu.edu.cn

**Keywords:** microfluidic biochips, high-level synthesis, fluidic port, volume management

## Abstract

Continuous-flow microfluidic biochips (CFMBs) automatically execute various bioassays by precisely controlling the transport of fluid samples, which is driven by pressure delivered through fluidic ports. High-level synthesis, as an important stage in the design flow of CFMBs, generates binding and scheduling solutions whose quality directly affects the efficiency of the execution of bioassays. Existing high-level synthesis methods perform numerous transport tasks concurrently to increase efficiency. However, fluidic ports cannot be shared between concurrently executing transport tasks, resulting in a large number of fluidic ports introduced by existing methods. Increasing the number of fluidic ports undermines the integration, reduces the reliability, and increases the manufacturing cost. In this paper, we propose a port-driven high-level synthesis method based on integer linear programming (ILP) called SlimPort, integrating the optimization of fluidic port number into high-level synthesis, which has never been considered in prior work. Meanwhile, to ensure bioassay correctness, volume management between devices with a non-fixed input/output ratio is realized. Additionally, two acceleration strategies for ILP, scheduling constraint reduction and upper boundary estimation of fluidic port number, are proposed to improve the efficiency of SlimPort. Experimental results from multiple benchmarks demonstrate that SlimPort leads to high assay execution efficiency and a low number of fluidic ports.

## 1. Introduction

Continuous-flow microfluidic biochips (CFMBs), also known as lab-on-a-chip systems, have attracted considerable research interest in both academia and industry over the past decade, owing to their high precision, high throughput, and low cost [1,2]. CFMBs have been developed for use in various bioassays, such as point-of-care diagnosis [3], cancer diagnostic [4,5], immunoassays [6,7], and air quality monitoring [8].

**Figure 1:**
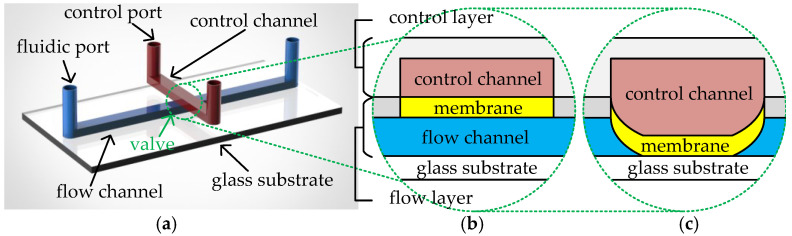
(**a**) Structure of a CFMB. Cross-section of (**b**) an open valve and (**c**) a closed valve.

A CFMB typically consists of two layers of polydimethylsiloxane (PDMS) material on a glass substrate [9], referred to as the control layer and the flow layer, respectively. Each of the two layers has its own channel network and is connected to the environment through its own ports, as shown in Figure 1a. Channels in the flow layer, also called flow channels, are connected to the external environment of the biochip through fluidic ports. Fluidic ports are divided into flow ports and waste ports. Flow ports are used for the input of samples/reagents and the injection of air pressure from external pressure sources. Waste ports collect the output fluid and release the air pressure. Channels in the control layer, also called control channels, are connected to the external pressure source through control ports. The overlapping region between a flow channel and a control channel forms an elastic PDMS membrane. The structure is capable of controlling fluid transport in the corresponding flow channel according to the air pressure in the control channel; hence, it is called a valve. Figure 1b,c illustrate the cross-section of the valve in the open and closed states, respectively. To close the valve, the high pressure, generated by an external pressure source injected through the control port, is conducted to the valve via the control channel. This causes the valve’s elastic membrane to be pushed downwards, sealing the corresponding flow channel, as shown in Figure 1c. When the high pressure is released, the valve’s elastic membrane returns to its original state and the flow channel reopens for fluid transfer.

**Figure 2:**
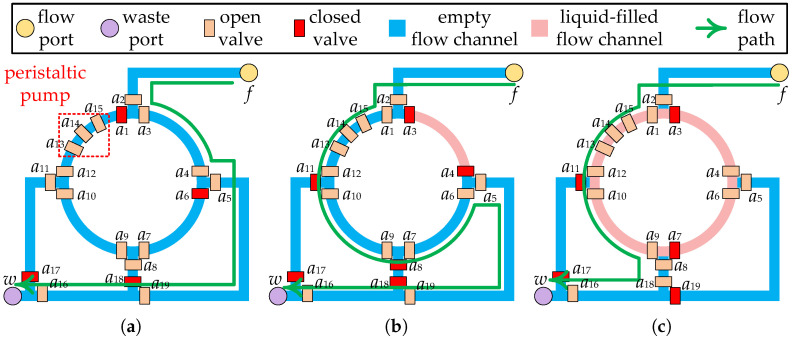
Part of the layout of the CFMB with a 4-segment rotary mixer. (**a**) Input fluid at 1/4 mixer capacity. (**b**) Input fluid at 3/4 mixer capacity. (**c**) Output fluid at 1/2 mixer capacity after mixing.

Complex microfluidic devices can be constructed with various combinations of valves, flow channels, and functional components (e.g., peristaltic pump, heating component, etc.) to perform a variety of biochemical operations [10]. For example, a four-segment rotary mixer, as shown in Figure 2a, consists of valves ($a_{1}–a_{12}$), ring channels, and a peristaltic pump ($a_{13}–a_{15}$), which has been widely used on CFMBs [11]. By orderly transport of samples/reagents between different devices, CFMBs are able to accomplish complex bioassays automatically. The transport of samples/reagents is driven by the pressure injected through the flow port, so flow channels are employed to construct a complete pressure propagation path, called a flow path. A flow path starts at a flow port, passes through the fluid start location and the fluid target location, and ends at a waste port. For example, the flow path for the transport task shown in Figure 2a is flow port $f\to a_{2}\to a_{3}\to a_{4}\to a_{5}\to a_{19}\to a_{16}\to$ waste port w. The fluid start location is the flow port, and the fluid target location is the channel between $a_{3}$ and $a_{4}$.

The feature size of the CFMB has been continuously reduced with the advancement of microfluidic manufacturing technology [12,13,14]. This allows CFMBs to perform more complex bioassays. Fluidigm’s 96.96 Dynamic Array, for instance, can run 9216 parallel polymerase chain reactions [15]. However, complex bioassays also mean that a large number of transport tasks need to be performed in parallel. To avoid pressure mutual interference and sample/reagent cross-contamination, separate flow paths need to be constructed for these parallel transport tasks. Each flow path requires a pair of fluidic ports, i.e., a flow port and a waste port. This leads to a rapid increase in fluidic ports. Previous work [16] has discussed the damage to the CFMB caused by integrating too many control ports. Analogous to the control ports, the fluidic ports are actually holes punched on the CFMB [17]. Too many holes punched on the CFMB not only take up chip area but also weaken the structural strength of the CFMB, making it more susceptible to physical failure [16]. In addition, fluidic ports also need to be connected to peripheral equipments (e.g., air pressure sources, etc.) for sample/reagent input and collection and to provide power for transport tasks. These peripheral pieces of equipment are still made for mechanical devices and are large in size [16]. These limit the potential of large-scale integration of CFMBs.

Over the past decade, considerable effort has been devoted to the design automation of CFMBs due to the high complexity of the CFMB architecture and bioassay protocol [18,19,20,21,22,23]. The architectural synthesis of CFMBs is usually divided into three major stages, high-level synthesis, physical design of the flow layer, and physical design of the control layer. High-level synthesis is an important step in the architectural synthesis of CFMBs. The goal of the high-level synthesis is to obtain binding and scheduling schemes that can be used as inputs for subsequent design steps, such as placement for devices, routing for flow path, etc. It is worth noting that the final chip layout is not determined during the high-level synthesis of biochips. As such, the binding and scheduling scheme generated in the high-level synthesis phase functions merely as an initial scheme that may be adapted in subsequent phases, but its quality directly affects the optimization of the subsequent phases. For example, the binding and scheduling scheme determines the devices to be placed and the connection relationships between them. A number of methods for high-level synthesis of CFMBs have been proposed. In [24], a list scheduling-based heuristic method is proposed to reduce the total application completion time. In [25], a graph-based approach, which formulates the high-level synthesis as a maximum clique finding problem, is proposed to accurately obtain an optimal binding and scheduling scheme. In [10], a component-oriented high-level synthesis method is proposed to improve the utilization of chip resources and to accommodate multiple types of microfluidic devices. In [26], a path-driven synthesis methodology is presented to integrate the actual fluid manipulations into both high-level synthesis and physical design. Volume management between devices and the removal of excess/waste fluids are introduced into the high-level synthesis to ensure the correctness of assay outcomes. In [16], a synthesis flow called MiniControl is proposed to generate chip architectures under strict constraints of control ports, where control-port minimization is considered systematically during the complete flow-layer design, including high-level synthesis. In [27], a high-level synthesis method considering fluid volume and channel storage is proposed to reduce the requirement of cache, thus ensuring the reliability of fluid caching under the distributed channel-storage architecture. In [28], the time constraints are introduced into the high-level synthesis to satisfy the real-time requirements of timing-sensitive bioassays. However, these methods do not take the fluidic port into account and perform numerous transportation tasks concurrently to increase efficiency. This results in a large demand for fluidic ports. The enormous potential for reducing the number of fluidic ports in high-level synthesis is neglected.

Due to the limitations of existing methods and the importance of reducing the number of ports, in this paper, we propose SlimPort, a high-level synthesis method for CFMBs. The major contributions are listed below.

- A reduction in the fluidic port number is incorporated into high-level synthesis for the first time, thereby reducing the fabrication cost and improving the reliability of CFMBs.
- We propose extended volume management to achieve volume constraints for devices with a non-fixed input/output ratio, ensuring the correctness of bioassay outcomes.
- We propose two acceleration strategies for integer linear programming (ILP), scheduling constraint reduction and upper boundary estimation of the port number, to reduce the complexity of the ILP model and speed up the time required to solve it.
- The effectiveness of SlimPort is demonstrated by experimental results on five real-world bioassays and five synthetic benchmarks.

## 2. High-Level Synthesis, Motivation, and Problem Formulation

**Figure 3:**
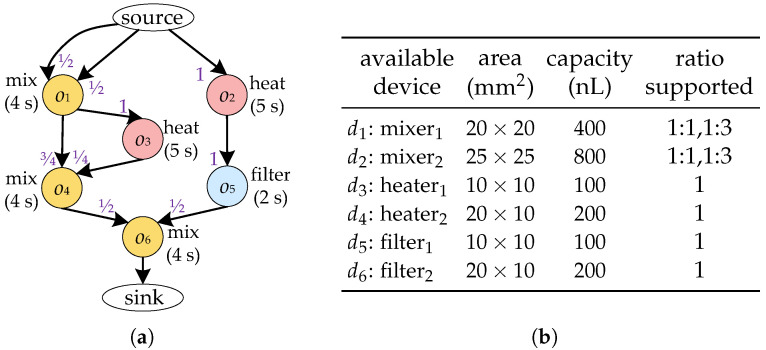
(**a**) Sequencing graph. (**b**) Device library.

High-level synthesis inputs include the bioassay protocol to be implemented and a device library to automatically generate an optimized binding and scheduling scheme for a given bioassay. As shown in Figure 3a, the bioassay protocol is modelled as a directed graph G(O,E), also known as a sequencing graph. Each node $o_{i}\in O$ represents a biochemical operation, such as heating and mixing, and is associated with a weight indicating its duration. Each edge $e_{i,j}\in E$ specifies the dependency between operations—i.e., operation $o_{i}$ is a parent node of $o_{j}$ in *G*. For the sake of simplicity, hereinafter we denote the device bound to operation $o_{i}$ by $d(o_{i})$. We use $f_{i,j}$ to represent the fluid from $d(o_{i})$ that needs to be transported to $d(o_{j})$. An edge $e_{i,j}\in E$ specifies the dependency between operations—i.e., operation $o_{i}$ is a parent node of $o_{j}$ in *G*. Each edge $e_{i,j}$ is associated with weights $r_{i,j}^{\mathrm{In}}$, which denotes the ratio of the volume f(i,j) to the capacity $d(o_{j})$ after inputting f(i,j) into $d(o_{j})$. A device library *D* used for realizing the execution of operations in *G*. Each device $d_{i}\in D$ is associated with its area and capacity, as shown in Figure 3b. The device library also provides the input/output ratios supported by each device. Given the sequencing graph of a bioassay and the corresponding device library, the high-level synthesis of CFMBs is usually divided into two major tasks: binding and scheduling.

### 2.1. Volume Management Between Devices with Non-Fixed Input/Output Ratio and Binding

The goal of binding is to select a specific device for the execution of each operation and to satisfy the volume constraint between devices [26]. The reaction chamber of the microfluidic device consists of one or more flow channel segments that are divided by valves [10]. For example, the reaction chamber of a four-segment rotary mixer consists of a ring flow channel divided into four flow channel segments by valves ($a_{1},a_{3},a_{4},a_{6},a_{7},a_{9},a_{10},a_{12}$), as shown in Figure 2a. The flow channel section of the device is filled with air rather than a vacuum in the initial state. All air should be removed from the device before the operation is carried out so that it does not affect the operation. At the start of the operation, it is also important to avoid leaving the air that drives the samples/reagents in the device. To prevent operations from failing, a minimum volume should be set for each input of a device to allow air to be expelled from the device while preventing outside air from entering.

Prior work has set a fixed minimum input volume limit for each device [26]. However, such setups cannot be applied on devices with non-fixed input/output ratios. Each device supports one or more input/output ratios, depending on the device architecture. For example, the four-segment rotary mixer can mix fluid in 1:1, 1:3, 1:1:2, or 1:1:1:1. As shown in Figure 2a,b, using the flow path as indicated by the green arrows, the mixer was successively input fluid with one-quarter and three-quarter mixer capacity, respectively. After performing a 1:3 ratio mixing operation, the fluid with one-half capacity of mixer can be removed using the flow path as shown by the green arrow in Figure 2c. It is possible to perform a 1:1 ratio mixing operation in the same mixer following the input of a fluid with one-half mixer capacity using the same flow path.

Due to the structural limitations of the device, the device cannot output/input fluid at any ratio. For example, the four-segment rotary mixer shown in Figure 2c is unable to remove all of the fluid from the ring channel with a single output procedure. Therefore, to ensure that the fluid volume input to the device satisfies the requirements of the operation $o_{i}$, $o_{i}$ should be bound to a device that supports the required input ratio. Moreover, the fluid volume output by $o_{i}$ should be determined based on the output ratio supported by $d(o_{i})$ to satisfy the volume constraint between devices. Consider the bioassay described in Figure 3a as an example. When $o_{1}$ and $o_{4}$ are bound to $d_{1}$, if $o_{i}$ is output at 1:1, the volume of $f_{1,4}$ is 200 nL, which is less than the volume of fluid required for $o_{4}$ (300 nL), violating the volume constraint between devices. In contrast, if $o_{1}$ is output at 1:3, $f_{1,4}$ has a volume of 300 nL and $f_{1,3}$ has a volume of 100 nL, satisfying the volume constraint.

### 2.2. Optimization of Fluidic Port Number and Scheduling

The goal of scheduling is to determine the start and end time for each operation and each transport task and to minimize bioassay completion time while satisfying given dependencies. Transport tasks in CFMBs can be divided into three categories: fluid transportation, excess fluid removal, and waste fluid removal [26]. The fluid transportation is the task of transporting samples/reagents between devices or between a port and a device. The excess fluid is cached in both ends of the device when the volume of input fluid is greater than the minimum input volume limit. To prevent excess fluid from interfering with other fluids in the CFMB, excess fluid removal should be performed before the next input to that device. Additionally, when the parent–child operations $o_{i}$ and $o_{j}$ are bound to the same device, if there are other inputs to the child operation $o_{j}$ and the volume of $f_{i,j}$ in $d(o_{j})$ is greater than the minimum input volume limit, then waste fluid removal should be performed to remove the fluid that is not needed anymore out of $d(o_{j})$.

**Figure 4:**
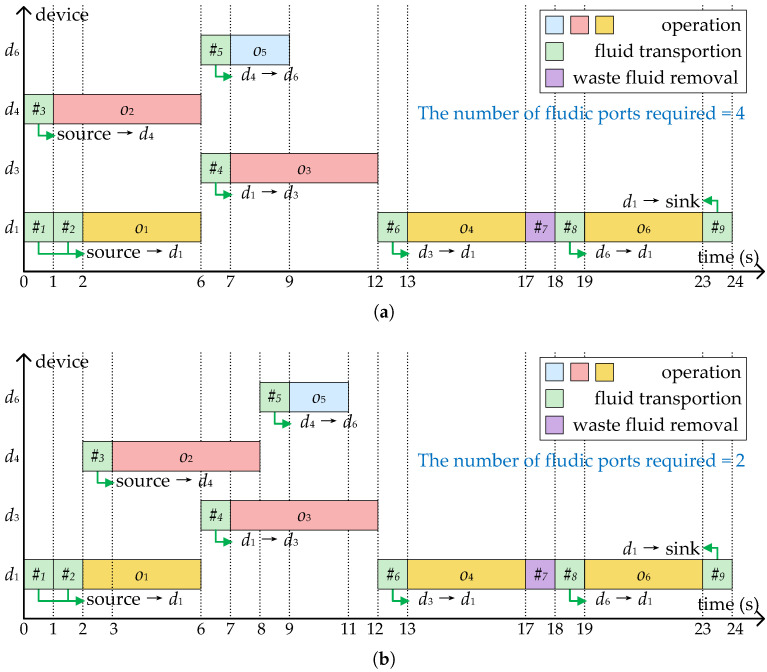
Two binding and scheduling schemes corresponding to Figure 3. (**a**) The scheme without considering the optimization of fluidic ports. (**b**) The scheme generated by SlimPort.

Figure 4a shows the scheduling results of the bioassay described in Figure 3a, where four devices are allocated to execute operations $o_{1}–o_{6}$, and the bioassay completed in 24 s with nine transport tasks. As mentioned before, each transport task needs to construct a flow path and associate it with a pair of flow ports. To avoid pressure mutual interference and sample/reagent cross-contamination, the same pair of flow ports cannot be shared between parallel transport tasks. In the scheduling scheme shown in Figure 4a, since ${\#}_{3}$ and ${\#}_{1}$, ${\#}_{5}$, and ${\#}_{4}$ are executed in parallel, at least two pairs of flow ports are required.

Correspondingly, Figure 4b shows a scheduling scheme considering the optimization of fluidic ports. By delaying the start of ${\#}_{3}$ and ${\#}_{5}$, there are no transport tasks that need to be performed in parallel throughout the bioassay. All transport tasks are allowed to share the same pair of fluidic ports—in other words, only two flow ports are required.

### 2.3. Problem Formulation

The high-level synthesis problem for CFMBs considered in this paper can be formulated as follows, based on the above analysis:

Inputs:

- A bioassay modeled as a sequencing graph G(O,E) with the type and duration of each operation, as well as the volume ratio of each input.
- A device library *D* with the area and capacity of each device and the input/output ratios supported by each device.

Outputs:

- A binding scheme satisfying the volume constraints between devices.
- The volume of fluid output from each operations.
- A scheduling scheme indicating the start and end time for each operation and each transport task.

Objectives: Minimizing the following indicators.

- The completion time of the bioassay.
- The number of fluidic ports required.
- The total area of devices employed.
- The volume of excess fluid and waste fluid.

## 3. Details of the Proposed Port-Driven High-Level Synthesis

In this section, we discuss the proposed SlimPort in detail. In the following, the high-level synthesis is formulated and solved as an integer linear programming (ILP) model to obtain an optimal binding and scheduling scheme. Moreover, we present the two acceleration strategies, constraint reduction and boundary estimation, both of which reduce the computational overhead of our approach. Table 1 lists symbols that are frequently used in SlimPort.

### 3.1. ILP Model Constructed by SlimPort

#### 3.1.1. Binding with Extended Volume Management

To ensure that each operation performs its function, each operation should be bound to a device. Thus, we have the following constraint:

(1) $$\begin{array}{c} \sum_{d_{k}\in D}b_{o_{i},d_{k}}=1,\forall o_{i}\in O, \end{array}$$

(2) $$\begin{array}{c} b_{o_{i},d_{k}}\leq \zeta_{o_{i},d_{k}},\forall o_{i}\in O,\forall d_{k}\in D, \end{array}$$

where $b_{o_{i},d_{k}}$ and $\zeta_{o_{i},d_{k}}$ are binary variables representing whether operation $o_{i}$ is bound to a device $d_{k}$ and whether $d_{k}$ can support the functions and input ratios required by $o_{i}$, respectively.

Each device supports one or more output ratios. When the device outputs at a specific ratio, we refer to the ratio between output fluid volume and device capacity as an output mode. We note all output modes supported by devices in the device library *D* as Q. When the device outputs in one output mode $Q_{l}\in Q$, we use $q_{l,h}$ to represent the *h*-th ratio between the output fluid volume and the device capacity. For example, the Q associated with the equipment library shown in Figure 3b is $\left\{1,\left\{\frac{1}{2},\frac{1}{2}\right\},\left\{\frac{1}{4},\frac{3}{4}\right\}\right\}$. Furthermore, the output modes supported by device $d_{1}$ are $Q_{2}=\left\{\frac{1}{2},\frac{1}{2}\right\}$ and $Q_{3}=\left\{\frac{1}{4},\frac{3}{4}\right\}$. The operation $o_{i}$ can be output in only one output mode, which can be formulated as follows:

(3) $$\begin{array}{c} \sum_{Q_{l}\in Q}\theta_{i,l}=1,\forall o_{i}\in O, \end{array}$$

(4) $$\begin{array}{c} \theta_{i,l}\leq \sum_{d_{k}\in D}\mathrm{Support}\left(d_{k},Q_{l}\right)\times b_{o_{i},d_{k}},\forall Q_{l}\in Q,\forall o_{i}\in O, \end{array}$$

where $\theta_{i,l}$ and $\mathrm{Support}(d_{k},P_{l})$ are binary variables representing whether $o_{i}$ outputs in the output mode $Q_{l}$ and whether $d_{k}$ can support the output mode $Q_{l}$.

$f_{i,j}$ should be transported from $d(o_{i})$ to $d(o_{j})$ for every $e_{i,j}\in E$. To determine the volume of $f_{i,j}$, we use a binary variable $\varpi_{i,j,l,h}$ to represent whether the ratio between the volume of $f_{i,j}$ and the capacity of $d(o_{i})$ is $q_{l,h}$. $\varpi_{i,j,l,h}$ can be further constrained as follows:

(5) $$\begin{array}{c} \sum_{q_{l,h}\in Q_{l}}\varpi_{i,j,l,h}=\theta_{i,l},\forall Q_{l}\in Q,\forall e_{i,j}\in E, \end{array}$$

(6) $$\begin{array}{c} \sum_{Q_{l}\in Q,q_{l,h}\in Q_{l}}\varpi_{i,j,l,h}=1,\forall e_{i,j}\in E. \end{array}$$

Then, the volume constraint between devices with a non-fixed input/output ratio can be formulated as follows:

(7) $$\begin{array}{c} \left(\sum_{Q_{l}\in Q,q_{l,h}\in Q_{l}}\varpi_{i,j,l,h}\times q_{l,h}\right)\times \left(\sum_{d_{k}\in D}b_{o_{i},d_{k}}\times c_{k}\right)-r_{i,j}^{\mathrm{In}}\times \sum_{d_{k}\in D}b_{o_{j},d_{k}}\times c_{k}=v_{i,j}\geq 0,\forall e_{i,j}\in E, \end{array}$$

where $c_{k}$ is the capacity of device $d_{k}$, $r_{i,j}^{\mathrm{In}}$ is the ratio of the volume f(i,j) to the capacity $d(o_{j})$ after f(i,j) is input to $d(o_{j})$, and $v_{i,j}$ is the volume of the excess fluid or the waste fluid generated by $f_{i,j}$.

#### 3.1.2. Scheduling with Optimization of Fluidic Ports

When transporting $f_{i,j}$ to $d(o_{i})$, we can optionally cache $f_{i,j}$ in storage and later transport it to $d(o_{i})$. We use $\phi_{i,j,1}$ and $\phi_{i,j,2}$ to represent the fluid transportation from $d(o_{i})$ to the storage and the fluid transportation to $d(o_{j})$. When $f_{i,j}$ is transported directly from $d(o_{i})$ to $d(o_{j})$, only the fluid transportation $\phi_{i,j,2}$ is performed. After completing the input of $f_{i,j}$ to $d(o_{j})$, if $v_{i,j}>0$, the excess liquids cached at both ends of $d(o_{i})$ should be removed. This results in excess fluid removal, denoted by $\phi_{i,j,l},l=3,4$. When $o_{i}$ and $o_{j}$ are bound to the same device and $f_{i,j}$ does not need to be cached in storage, it is unnecessary to perform $\phi_{i,j,l},l=1,2$. If $v_{i,j}>0$ in this case, there is waste fluid in $d(o_{j})$ that needs to be removed. This results in waste fluid removal, denoted by $\phi_{i,j,5}$. Thus, we have the following constraint:

(8) $$\begin{array}{c} t_{\phi_{i,j,l}}^{e}-t_{\phi_{i,j,l}}^{s}=T_{\phi }\times \eta_{i,j,l},\forall e_{i,j}\in E,l\in \left[1,5\right], \end{array}$$

where $t_{\phi_{i,j,l}}^{e}$ and $t_{\phi_{i,j,l}}^{s}$ are the end time and the start time of $\phi_{i,j,l},l\in \left[1,5\right]$, respectively. $T_{\phi }$ represents the duration of a transport task. $\eta_{i,j,l},l\in \left[1,5\right]$ is a binary variable representing whether $\phi_{i,j,l}$ should be performed. $\eta_{i,j,l}$ can be further constrained as follows:

(9) $$\begin{array}{c} \eta_{i,j,1}\leq \eta_{i,j,2},\forall e_{i,j}\in E, \end{array}$$

(10) $$\begin{array}{c} 1-\sum_{d_{k}\in D}z_{i,j,k}\leq \eta_{i,j,2}\leq \eta_{i,j,1}+1-\sum_{d_{k}\in D}z_{i,j,k},\forall e_{i,j}\in E, \end{array}$$

(11) $$\begin{array}{c} \tau_{i,j}\times \epsilon \leq v_{i,j}\leq \tau_{i,j}\times M,\forall e_{i,j}\in E, \end{array}$$

(12) $$\begin{array}{c} 2\times \eta_{i,j,l}\leq \tau_{i,j}+\eta_{i,j,1}\leq 1+\eta_{i,j,l},\forall e_{i,j}\in E,l=3,4, \end{array}$$

(13) $$\begin{array}{c} 2\times \eta_{i,j,5}\leq \tau_{i,j}+1-\eta_{i,j,1}\leq 1+\eta_{i,j,5},\forall e_{i,j}\in E, \end{array}$$

where $\tau_{i,j}$ and $z_{i,j,k}$ are binary variables representing whether $v_{i,j}$ is greater than 0 and whether $o_{i}$ and $o_{j}$ are bound to $d_{k}$, respectively. $z_{i,j,k}$ can be further constrained as follows:

(14) $$\begin{array}{c} 2\times z_{i,j,k}\leq b_{o_{i},d_{k}}+b_{o_{i},d_{k}}\leq z_{i,j,k}+1,\forall o_{i},o_{j}\in O,\forall d_{k}\in D. \end{array}$$

The fluid transportation $\phi_{i,j,2}$ should not be started until the fluid transportation $\phi_{i,j,1}$ has been completed. Moreover, the excess fluid removal $t_{i,j,l}^{s},l=3,4$ should be performed after transportation $\phi_{i,j,2}$ has finished. Then, the waste fluid removal $t_{i,j,5}^{s}$ is performed. Thus, we have the following constraint:

(15) $$\begin{array}{c} t_{\phi_{i,j,1}}^{e}\leq t_{\phi_{i,j,2}}^{s},\forall e_{i,j}\in E, \end{array}$$

(16) $$\begin{array}{c} t_{i,j,2}^{e}\leq t_{i,j,l}^{s},\forall e_{i,j}\in E,l=3,4. \end{array}$$

The inputs of $o_{j}$ should be loaded separately, which can be constrained as follows:

(17) $$\begin{array}{c} \begin{array}{cc} t_{\phi_{i,j,l}}^{e} & \leq t_{\phi_{h,j,2}}^{s}+\left(1-\psi_{i,h,j}\right)\times M\\ t_{\phi_{h,j,l}}^{e} & \leq t_{\phi_{i,j,2}}^{s}+\psi_{i,h,j}\times M \end{array},\forall e_{i,j},e_{h,j}\in E,l=3,4, \end{array}$$

where $\psi_{i,h,j}$ is a binary variable representing the order of the inputs from $o_{i}$ and $o_{h}$ to $o_{j}$ and *M* is a very large constant for transforming two situations indicated by $\psi_{i,h,j}$ into linear constraints.

The operation can only be performed after all inputs have been completed. The operation should then last for the specified time to implement the corresponding functionality. Thus, we have the following constraint:

(18) $$\begin{array}{c} t_{\phi_{i,j,2}}^{e}\leq t_{o_{j}}^{s},\forall e_{i,j}\in E,t_{o_{j}}^{e}-t_{o_{j}}^{s}=T\left(o_{j}\right),\forall o_{j}\in O, \end{array}$$

where $t_{o_{j}}^{e}$ and $t_{o_{j}}^{s}$ are the end time and the start time of $o_{j}$, respectively, and $T(o_{j})$ is the execution time of $o_{j}$.

Outputs of $o_{j}$ should only be executed after the operation $o_{j}$ has been completed and the outputs of $o_{j}$ should be removed separately. Thus, we have the following constraint:

(19) $$\begin{array}{c} t_{o_{j}}^{e}\leq t_{\phi_{j,h,1}}^{s},\forall e_{j,h}\in E, \end{array}$$

(20) $$\begin{array}{c} t_{\phi_{j,i,1}}^{e}\leq t_{\phi_{j,h,1}}^{s}+\left(1-\xi_{i,h,j}\right)\times M,\forall e_{j,i},e_{j,h}\in E, \end{array}$$

(21) $$\begin{array}{c} t_{\phi_{j,i,2}}^{e}\leq t_{\phi_{j,h,1}}^{s}+\left(1-\xi_{i,h,j}+\eta_{j,i,1}\right)\times M,\forall e_{j,i},e_{j,h}\in E, \end{array}$$

(22) $$\begin{array}{c} t_{\phi_{j,h,1}}^{e}\leq t_{\phi_{j,i,1}}^{s}+\xi_{i,h,j}\times M,\forall e_{j,i},e_{j,h}\in E, \end{array}$$

(23) $$\begin{array}{c} t_{\phi_{j,h,2}}^{e}\leq t_{\phi_{j,i,1}}^{s}+\left(\xi_{i,h,j}+\eta_{j,h,1}\right)\times M,\forall e_{j,i},e_{j,h}\in E, \end{array}$$

where $\xi_{i,h,j}$ is a binary variable representing the order of the outputs from $o_{j}$ to $o_{i}$ and $o_{h}$.

Additionally, without loss of correctness, we make $t_{i,j,5}^{e}$ equal to the time at which all fluids generated by $o_{i}$ leave $d(o_{i})$ via the following constraints:

(24) $$\begin{array}{c} t_{\phi_{i,j,2}}^{s}\leq t_{\phi_{i,j,1}}^{s}+\eta_{i,j,1}\times M,\forall e_{i,j}\in E, \end{array}$$

(25) $$\begin{array}{c} t_{i,j,1}^{e}\leq t_{i,j,5}^{s},\forall e_{i,j}\in E, \end{array}$$

(26) $$\begin{array}{c} t_{i,j,2}^{e}\leq t_{i,j,5}^{s}+\eta_{i,j,1}\times M,\forall e_{i,j}\in E. \end{array}$$

When two operations are bound to the same component, all fluids generated by one operation should be removed before the other operation can start executing the inputs, constrained as follows:

(27) $$\begin{array}{c} \begin{array}{cc} t_{\phi_{i,j^{\prime },5}}^{e} & \leq t_{\phi_{i^{\prime },j,2}}^{s}+\left(2-\sum_{d_{k}\in D}z_{i,j,k}-\sigma_{i,j}\right)\times M,\forall e_{i,j^{\prime }},e_{i^{\prime },j}\in E\\ t_{\phi_{j,i^{\prime },5}}^{e} & \leq t_{\phi_{j^{\prime },i,2}}^{s}+\left(1-\sum_{d_{k}\in D}z_{i,j,k}+\sigma_{i,j}\right)\times M,\forall e_{j,i^{\prime }},e_{j^{\prime },i}\in E \end{array},\forall o_{i},o_{j}\in O, \end{array}$$

where $\sigma_{i,j}$ is a binary variable representing the order of $o_{i}$ and $o_{j}$.

We assume that there is at most one storage on the CFMBs. Due to the bandwidth limitations of the storage, all cache inputs and cache outputs should be separated.

(28) $$\begin{array}{c} \begin{array}{c} t_{\phi_{i,j,l}}^{e}\leq t_{\phi_{i^{\prime },j^{\prime },l^{\prime }}}^{s}+\left(3-\eta_{i,j,1}-\eta_{i^{\prime },j^{\prime },1}-\rho_{\phi_{i,j,l},\phi_{i^{\prime },j^{\prime },l^{\prime }}}\right)\times M\\ t_{\phi_{i^{\prime },j^{\prime },l^{\prime }}}^{e}\leq t_{\phi_{i,j,l}}^{s}+\left(2-\eta_{i,j,1}-\eta_{i^{\prime },j^{\prime },1}+\rho_{\phi_{i,j,l},\phi_{i^{\prime },j^{\prime },l^{\prime }}}\right)\times M \end{array},\forall e_{i,j},e_{i^{\prime },j^{\prime }}\in E,l=1,2,l^{\prime }=1,2, \end{array}$$

where $\rho_{\phi_{i,j,l},\phi_{i^{\prime },j^{\prime },l^{\prime }}}$ is a binary variable representing the order of $\phi_{i,j,l}$ and $\phi_{i^{\prime },j^{\prime },l^{\prime }}$.

As previously mentioned, each transport task should be associated with a pair of fluidic ports to provide pressure. We use a binary variable $b_{\phi_{i,j,l},p_{k}}$ to represent whether the $\phi_{i,j,l}$ is bound to fluidic port pair $p_{k}$. We have

(29) $$\begin{array}{c} \sum_{p_{k}\in P}b_{\phi_{i,j,l},p_{k}}=\eta_{i,j,l},\forall e_{i,j}\in E,l\in \left[1,5\right], \end{array}$$

where *P* is the set of fluidic port pairs.

Transport tasks bound to the same pair of fluid ports cannot be executed in parallel, constrained as follows:

(30) $$\begin{array}{c} \begin{array}{c} t_{\phi_{i,j,l}}^{e}\leq t_{\phi_{i^{\prime },j^{\prime },l^{\prime }}}^{s}+\left(3-b_{\phi_{i,j,l},p_{k}}-b_{\phi_{i^{\prime },j^{\prime },l^{\prime }},p_{k}}-\rho_{\phi_{i,j,l},\phi_{i^{\prime },j^{\prime },l^{\prime }}}\right)\times M\\ t_{\phi_{i^{\prime },j^{\prime },l^{\prime }}}^{e}\leq t_{\phi_{i,j,l}}^{s}+\left(2-b_{\phi_{i,j,l},p_{k}}-b_{\phi_{i^{\prime },j^{\prime },l^{\prime }},p_{k}}+\rho_{\phi_{i,j,l},\phi_{i^{\prime },j^{\prime },l^{\prime }}}\right)\times M \end{array},\forall e_{i,j},e_{i^{\prime },j^{\prime }}\in E,l,l^{\prime }\in \left[1,5\right],p_{k}\in P, \end{array}$$

where $\rho_{\phi_{i,j,l},\phi_{i^{\prime },j^{\prime },l^{\prime }}}$ is a binary variable representing the order of $\phi_{i,j,l}$ and $\phi_{i^{\prime },j^{\prime },l^{\prime }}$.

#### 3.1.3. Optimization Objective

Once all operations and transport tasks have been completed, the bioassay is complete, constrained as follows:

(31) $$\begin{array}{c} t_{o_{i}}^{e}\leq T^{e},\forall o_{i}\in O \end{array}$$

(32) $$\begin{array}{c} t_{\phi_{i,j,l}}^{e}\leq T^{e},\forall e_{i,j}\in E,l\in \left[1,5\right], \end{array}$$

where $T^{e}$ is the completion time of the bioassay.

We use binary variables ${\mathrm{use}}_{p_{k}}$ and ${\mathrm{use}}_{d_{k}}$ to represent whether $p_{k}$ and $d_{k}$ are allocated, respectively, constrained as follows:

(33) $$\begin{array}{c} b_{\phi_{i,j,l},p_{k}}\leq {\mathrm{use}}_{p_{k}},\forall e_{i,j}\in E,l\in \left[1,5\right],\forall p_{k}\in P, \end{array}$$

(34) $$\begin{array}{c} \eta_{i,j,1}\leq {\mathrm{use}}_{d_{\mathrm{storage}}},\forall e_{i,j}\in E, \end{array}$$

(35) $$\begin{array}{c} b_{o_{i},d_{k}}\leq {\mathrm{use}}_{d_{k}},\forall o_{i}\in O,\forall d_{k}\in D, \end{array}$$

where $d_{\mathrm{storage}}$ represents the storage in the device library.

Finally, an efficient high-level scheme can be generated by solving the following problem:

(36) $$\begin{array}{c} \mathrm{minimize}\;\alpha \times T^{e}+\beta \times \sum_{p_{k}\in P}{\mathrm{use}}_{p_{k}}+\gamma \times \sum_{d_{k}\in D}\left({\mathrm{use}}_{d_{k}}\times S_{k}\right)+\delta \times \sum_{e_{i,j}\in E}v_{i,j}, \end{array}$$

(37) $$\begin{array}{c} \mathrm{subject}\;\mathrm{to}\;(1)–(35), \end{array}$$

where α, β, γ, and δ are three weighting factors and $S_{k}$ is the area of $d_{k}$.

### 3.2. Acceleration Strategies

To ensure the efficiency of SlimPort, two acceleration strategies, scheduling constraint reduction and upper boundary estimation of the port number, are proposed in this paper. The complexity of an ILP model is related to the number of its constraints and variables. The main idea of our proposed acceleration strategies is to reduce the number of constraints and variables that need to be constructed using the a priori knowledge provided by the inputs to achieve the acceleration.

#### 3.2.1. Scheduling Constraint Reduction

The constraint given in (27) restricts the order between two-by-two operations. For the sake of simplicity, we define the upstream operations of $o_{i}$ as all operations on the path from the source to $o_{i}$. Similarly, the downstream operations of $o_{i}$ are all operations on the path from $o_{j}$ to the source. Based on the dependencies in the sequencing graph, it is known that the upstream operations of $o_{i}$ should be completed before $o_{i}$ is executed and the downstream operations of $o_{i}$ should be executed after $o_{i}$ is completed. For operations of known order, we remove them from Constraint (27), thus achieving the reduction of Constraint (27).

Constraints (28) and (30) restrict the order between two-by-two transport tasks. For the sake of simplicity, we define the upstream edges of $e_{i,j}$ as all edges on the path from the source to $o_{i}$. Similarly, the downstream edges of $e_{i,j}$ are all operations on the path from $o_{j}$ to the source. Based on the dependencies in the sequencing graph, it is known that if $e_{i^{\prime },j^{\prime }}$ is the upstream edge of $e_{i,j}$, $\phi_{i^{\prime },j^{\prime },l^{\prime }},l^{\prime }\in \left[1,5\right]$ should be completed before $\phi_{i,j,l},l\in \left[1,5\right]$ is executed. And if $e_{i^{\prime },j^{\prime }}$ is the downstream edge of $e_{i,j}$, $\phi_{i^{\prime },j^{\prime },l^{\prime }},l^{\prime }\in \left[1,5\right]$ should be executed after $\phi_{i,j,l},l\in \left[1,5\right]$ is completed. For transport tasks of known order, we remove them from Constraints (28) and (30), thus achieving the reduction of Constraints (28) and (30).

#### 3.2.2. Upper Boundary Estimation of Fluidic Port Number

The number of variables $b_{\phi_{i,j,l},p_{k}}$ and the number of Constraints (29) and (30) are related to the number of ports in *P*. To ensure the correctness while reducing the complexity of the ILP model, we estimate the upper bound on the number of fluid ports using Algorithm 1. For the sake of simplicity, we define $e_{i,j}$ and $e_{i^{\prime },j^{\prime }}$ such that they are compatible when (1) $e_{i^{\prime },j^{\prime }}$ is not the upstream/downstream edge of $e_{i,j}$ and (2) $i\neq i^{\prime }\wedge j\neq j^{\prime }\wedge i\neq j^{\prime }\wedge j\neq i^{\prime }$. Given the sequencing graph G(O,E) and the component library *D*, we first construct an edge-compatible graph. A node of the edge-compatible graph is represented as $e_{i,j}\in E$. The edge between $e_{i,j}$ and $e_{i^{\prime },j^{\prime }}$ represents that $e_{i,j}$ and $e_{i^{\prime },j^{\prime }}$ are compatible. We then obtain all maximal cliques in the edge-compatible graph via the Bron–Kerbosch algorithm [29]. Finally, we traverse all maximal clusters and obtain the upper bound on the number of fluid ports.
**Algorithm 1:** Upper Boundary Estimation of Fluidic Port Number **Input**: The sequencing graph G(O,E) and the device library *D* **Output**: The upper boundary of fluidic port number $N_{port}^{upper}$ **_1_ for** *each*
$e_{i,j}\in E$
**do** **_2_**  $\Psi_{i,j}=\emptyset$; **_3_**  Add all $e_{i,h}\in E$ into $\Psi_{i,j}$; **_4_**  Add all $e_{h,j}\in E$ into $\Psi_{i,j}$; **_5_**  Initialize a stack S and push all $e_{j,h}\in E$ into S; **_6_  while** S≠∅ **do** **_7_**    
$e_{i^{\prime },j^{\prime }}=S.\mathrm{pop}\left(\right)$; **_8_**    Push all $e_{j^{\prime },h^{\prime }}\in E$ into S; **_9_**    Add all $e_{j^{\prime },h^{\prime }}\in E$ into $\Psi_{i,j}$; **_10_  end while** **_11_ end for** **_12_** Construct an edge-compatible graph with each node representing any edge in *E*, and if $e_{i,j}\notin \Psi_{i^{\prime },j^{\prime }}\wedge e_{i^{\prime },j^{\prime }}\notin \Psi_{i,j}$, then connect the nodes corresponding to these $e_{i,j}$ and $e_{i^{\prime },j^{\prime }}$ with a single edge; **_13_** Get all maximal cliques C in the edge-compatible graph; **_14_ for** *each*
$c_{i}\in C$ **do** **_15_**  Get the parent operations corresponding to the edge nodes in $c_{i}$ and count the number of operations of each type, denoted as $n_{1}^{s},n_{2}^{s},\ldots ,n_{N_{type}}^{s}$, where $N_{type}$ is the type number of all device in *D*; **_16_**  Get the child operations corresponding to the edge nodes in $c_{i}$ and count the number of operations of each type, denoted as $n_{1}^{e},n_{2}^{e},\ldots ,n_{N_{type}}^{e}$; **_17_**  $N_{c_{i}}=\min\left\{\sum_{i=0}^{N_{type}}\min\{n_{i}^{s},n_{i}^{d}\},\sum_{i=0}^{N_{type}}\min\{n_{i}^{e},n_{i}^{d}\}\right\}$, where $n_{i}^{d}$ is the number of devices with type i; **_18_ end for** **_19_** $N_{port}^{upper}=2*max_{i\in C}N_{i}$

## 4. Experimental Results

The proposed SlimPort was implemented in Python 3.11.11 and tested on a PC with 2.50 GHz CPU and 8 GB memory. The solver Gurobi was employed to solve the ILP model proposed in Section 3. As shown in Table 2, there are ten benchmarks to verify the performance of SlimPort. Five are synthetic benchmarks and the rest are real-world biochemical applications [26]. Parameters |O|,|E|, and |device| in Table 2 are the operation number and the edge number of the sequencing graph and the number of corresponding devices provided, respectively. The experimental parameters are set as follows: α=0.6, β=0.1, γ=0.002, and δ=0.001.

### 4.1. Validation of the Proposed SlimPort

Since there is no existing work considering the volume management between devices with a non-fixed input/output ratio, we implemented another method, called PD, by modifying the high-level synthesis method in [26], to verify the performance of the proposed SlimPort.

We ran both algorithms on the aforementioned benchmarks. Table 3 shows comparison results between SlimPort and PD, where columns $T^{e}$, $V_{r}$, and $N_{p}$ are the completion time of the bioassay, the volume of excess/waste fluid, and the number of fluidic ports required, respectively. Moreover, the column “Imp (%)” provides the relative improvements of SlimPort over PD.

**Figure 5:**
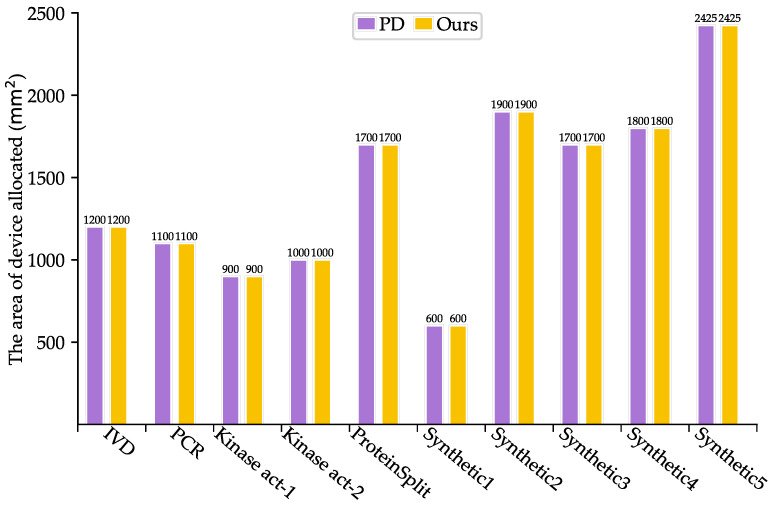
Comparison results between PD and SlimPort in terms of the area of devices allocated.

**Figure 6:**
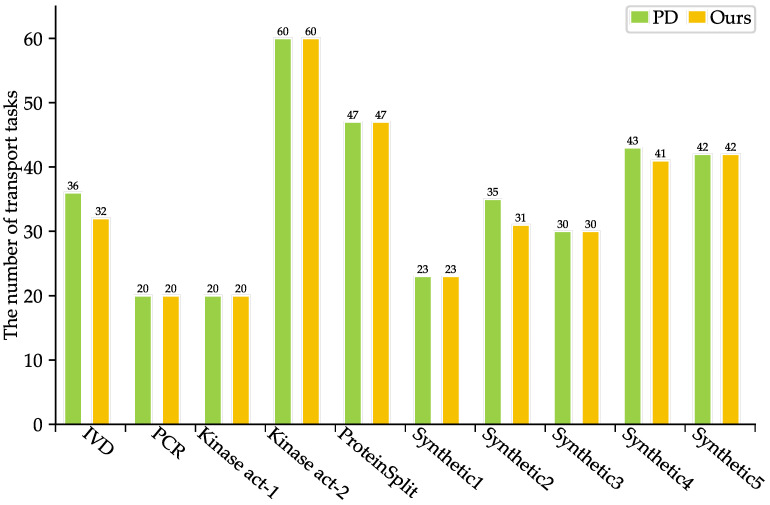
Comparison results between PD and SlimPort in terms of the number of transport tasks.

As shown in Table 3, SlimPort achieves a 33.3–66.7% reduction in terms of the number of fluidic ports required, with an average reduction of 51.67%, while maintaining the optimal solution in terms of bioassay completion time. Moreover, the volume of excess/waste fluid is reduced by 12.54%, thus reducing reagent consumption. As shown in Figure 5 and Figure 6, SlimPort maintains the optimal solution in terms of the area of devices allocated and improves the number of transport tasks by 2.72% on average compared with PD. PD takes the bioassay completion time and the area of devices allocated as the optimization objective and achieves the optimal solution. SlimPort is able to optimize the number of fluidic ports required, the volume of excess/waste fluid, and the number of transport tasks and maintain the optimal bioassay completion time and the area of devices allocated.

### 4.2. Validation of the Acceleration Strategies

**Figure 7:**
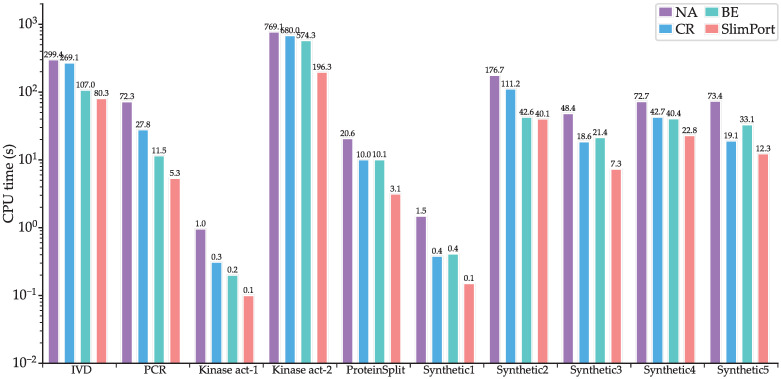
CPU times of NA, CR, BE, and SlimPort.

To verify the effectiveness of the proposed acceleration strategies, we implemented three other methods, NA, CR, and BE, where NA is based on the ILP model without acceleration, CR is based on the ILP model with scheduling constraint reduction, and BE is based on the ILP model with upper boundary estimation of the fluidic port number. Figure 7 shows CPU times of NA, CR, BE, and SlimPort. It can be seen that the computational efficiency improved for SlimPort across all the benchmarks compared with NA, CR, and BE. Compared with NA, CR achieves a maximum speedup of 3.8× with an average speedup of 2.3×, BE achieves a maximum speedup of 6.2× with an average speedup of 3.1×, and SlimPort achieves a maximum speedup of 13.5× with an average speedup of 6.7×. The acceleration capability of the acceleration strategies is demonstrated by the results above.

## 5. Conclusions

In this paper, we proposed a port-driven high-level synthesis method, called SlimPort, for CFMBs. Compared with the previous methods, SlimPort for the first time integrates the optimization of fluidic port number into the high-level synthesis. Volume management between devices with a non-fixed input/output ratio is also taken into account by SlimPort. Furthermore, two acceleration strategies were proposed to improve the overall performance of SlimPort: reducing scheduling constraints and estimating the upper bound of the fluidic port number. Experimental results on both real-life chip applications and synthetic benchmarks confirm the effectiveness of SlimPort.

Furthermore, in the future, we intend to implement a physical design method, which will be based on the results of the proposed SlimPort, for the co-design of the flow and control layers and flow path planning. The objective of this method is to generate an efficient chip architecture and optimize the number of fluidic and control ports together.

## Figures and Tables

**Table 1:** Symbols frequently used in SlimPort.

$T(o_{j})$: The execution time of $o_{j}$. $b_{o_{i},d_{k}}$: A binary variable representing whether operation $o_{i}$ is bound to a device $d_{k}$. $\zeta_{o_{i},d_{k}}$: A binary variable representing whether $d_{k}$ can support the functions and input ratios required by $o_{i}$. $f_{i,j}$: The fluid from $d(o_{i})$ that needs to be transported to $d(o_{j})$. $d(o_{i})$: The device bound to operation $o_{i}$. Q: All output modes supported by devices in the device library *D*. $Q_{l}$: The *l*-th output modes supported by devices in the device library *D*. $q_{l,h}$: The *h*-th ratio between the output fluid volume and the device capacity when the device outputs in one output mode $P_{l}$. $\theta_{i,l}$: A binary variable representing whether $o_{i}$ outputs in the output mode $P_{l}$. $\mathrm{Support}(d_{k},P_{l})$: A binary variable representing whether $d_{k}$ can support the output mode $P_{l}$. $\varpi_{i,j,l,h}$: A binary variable representing whether the ratio between the volume of $f_{i,j}$ and the capacity of $d(o_{i})$ is $p_{l,h}$. $r_{i,j}^{\mathrm{In}}$: The ratio of the volume f(i,j) to the capacity $d(o_{j})$ after f(i,j) is input to $d(o_{j})$. $c_{k}$: The capacity of device $d_{k}$. $v_{i,j}$: The volume of the excess fluid or the waste fluid generated by $f_{i,j}$. $\phi_{i,j,l},l\in \left[1,5\right]$: (1) $\phi_{i,j,1}$ represents the fluid transportation from $d(o_{i})$ to storage. (2) $\phi_{i,j,2}$ represents the fluid transportation to $d(o_{j})$. (3) $\phi_{i,j,l},l=3,4$ represents the excess fluid removal after $\phi_{i,j,2}$. (4) $\phi_{i,j,5}$ represents the waste fluid removal of $d_{i}$ when transporting $f_{i,j}$ to $d_{j}$. $t_{\phi_{i,j,l}}^{s},t_{\phi_{i,j,l}}^{e}$: The start time/end time of $\phi_{i,j,l}$. $\eta_{i,j,l}$: A binary variable representing whether $\phi_{i,j,l}$ should be performed. $t_{o_{i}}^{s},t_{o_{i}}^{e}$: The start time/end time of $o_{i}$. $\tau_{i,j}$: A binary variable representing whether $v_{i,j}$ is greater than 0. $z_{i,j,k}$: A binary variable representing whether $o_{i}$ and $o_{j}$ are bound to $d_{k}$. $\psi_{i,h,j}$: A binary variable representing the order of the inputs from $o_{i}$ and $o_{h}$ to $o_{j}$. *M*: A very large constant. $\xi_{i,h,j}$: A binary variable representing the order of the outputs from $o_{j}$ to $o_{i}$ and $o_{h}$. $\sigma_{i,j}$: A binary variable representing the order of $o_{i}$ and $o_{j}$. $b_{\phi_{i,j,l},p_{k}}$: A binary variable representing whether the $\phi_{i,j,l}$ is bound to fluidic port pair $p_{k}$. $\rho_{\phi_{i,j,l},\phi_{i^{\prime },j^{\prime },l^{\prime }}}$: A binary variable representing the order of $\phi_{i,j,l}$ and $\phi_{i^{\prime },j^{\prime },l^{\prime }}$. $T^{e}$: The completion time of the bioassay. $S_{k}$: The area of $d_{k}$. ${\mathrm{use}}_{d_{k}},{\mathrm{use}}_{p_{k}}$: binary variables representing whether $p_{k}$ and $d_{k}$ are allocated, respectively.

**Table 2:** Details of benchmarks used in experiments.

Benchmarks				
|O|/(|mixer|, |heater|, |filter|, |separator|, |detector|, |storage|)/|E|				
PCR	IVD	ProteinSplit	Kinase act-1	Kinase act-2
7/(4, 0, 0, 0, 0, 1)/15	12/(4, 0, 0, 0, 4, 1)/24	14/(4, 0, 0, 3, 3, 1)/27	4/(4, 0, 0, 4, 0, 1)/16	12/(4, 0, 0, 4, 0, 1)/48
Synthetic1	Synthetic2	Synthetic3	Synthetic4	Synthetic5
10/(4, 2, 3, 0, 2, 1)/15	15/(4, 3, 2, 0, 3, 1)/21	20/(4, 4, 3, 4, 2, 1)/28	25/(4, 4, 3, 0, 2, 1)/33	30/(4, 3, 3, 2, 4, 1)/42

**Table 3:** Comparison results between SlimPort and PD in terms of the completion time of the bioassay, the volume of excess/waste fluid, and the number of fluidic ports required.

Benchmarks	$T^{e}$ (s)			$V_{r}$ (nL)			$N_{p}$		
	PD	Ours	Imp (%)	PD	Ours	Imp (%)	PD	Ours	Imp (%)
IVD	29	29	0.00	1200	1000	16.67	12	4	66.67
PCR	24	24	0.00	1000	800	20.00	6	2	66.67
Kinase act-1	35	35	0.00	600	600	0.00	4	2	50.00
Kinase act-2	52	52	0.00	1300	900	30.77	12	4	66.67
ProteinSplit	88	88	0.00	2300	2300	0.00	8	4	50.00
Synthetic1	33	33	0.00	1200	1200	0.00	6	4	33.33
Synthetic2	39	39	0.00	1600	1100	31.25	6	4	33.33
Synthetic3	53	53	0.00	200	200	0.00	4	2	50.00
Synthetic4	54	54	0.00	1500	1100	26.67	12	4	66.67
Synthetic5	67	67	0.00	400	400	0.00	6	4	33.33
Average	-		0.00	-		12.54	-		51.67

## Data Availability

Dataset available on request from the authors.

## Abbreviations

The following abbreviations are used in this manuscript:

PDMS	Polydimethylsiloxane
CFMB	Continuous-flow microfluidic biochip
ILP	Integer linear programming
